# Predictors of Seizure Recurrence after Acute Symptomatic Seizures in Ischemic Stroke Patients

**DOI:** 10.1155/2019/8183921

**Published:** 2019-10-31

**Authors:** Pravin George, Vineet Punia, Prashant A. Natteru, Stephen Hantus, Christopher Newey

**Affiliations:** ^1^Cleveland Clinic, Department of Neurology, 9500 Euclid Avenue, Cleveland, OH 44195, USA; ^2^University of Mississippi Medical Center, 2500 North State Street, Jackson, MS 39216-4505, USA

## Abstract

**Purpose:**

Seizure is a well-recognized complication of both remote and acute ischemic strokes. Predictors of seizure recurrence and epilepsy in patients with ischemic stroke who develop acute symptomatic seizures (ASyS) on continuous electroencephalography (cEEG) have not been well studied.

**Methods:**

We present a five-year retrospective study of acute and remote ischemic stroke patients who developed ASyS on cEEG. We then identified risk factors for the development of seizure recurrence.

**Results:**

Sixty-five patients with ischemic stroke and ASyS were identified and reviewed. All ASyS were noted to be nonconvulsive seizures. Clinical recurrence of seizures was identified in 19 of these patients (29.2%) at follow-up. Rate of seizure recurrence was higher in remote ischemic stroke patients (84.2%), compared to acute ischemic stroke patients (15.8%, *p* = 0.0116, OR 0.17, 95% CI 0.049–0.65). Sharp waves/spikes on follow-up EEG significantly correlated with seizure recurrence (*p* = 0.006, OR 0, 95% CI 0–0.3926). Patients discharged on ≥3 antiepileptic drugs (AEDs) were at a higher risk of having seizure recurrence (*p* = 0.0015, OR 0.05, 95% CI 0.0089–0.37).

**Conclusion:**

We identified risk factors of seizure recurrence in patients with ASyS as remote ischemic stroke, requiring multiple AEDs, and the presence of sharp waves on follow-up EEG. This study highlights the usefulness of cEEG in evaluating patients with acute or remote strokes.

## 1. Introduction

Seizures are well-recognized complications of ischemic strokes, especially in older adults [1, 2]. Depending on the type, location, and severity of stroke, about 10% of patients will suffer from an acute symptomatic seizure (ASyS) [3]. 3–30% of the patients with stroke develop poststroke epilepsy [4]. The risk of subsequent development of epilepsy is highest in patients with remote ischemic stroke (i.e., >2 weeks from ischemic stroke onset) [5–8].

The pathophysiology for acute and remote poststroke seizures is thought to be distinct. Seizures due to acute stroke (i.e., ≤2 weeks from ischemic stroke onset) are probably due to the excitotoxic increased extracellular concentration of glutamate, causing secondary neuronal injury, along with structural and functional GABAergic impairment [9, 10]. Remote poststroke seizures are likely due to gliosis with changes in membrane properties, deafferentation, collateral sprouting, and neuronal loss resulting in hyperexcitability [11, 12].

Seizures profoundly impact the outcomes of ischemic stroke patients. The metabolic demand from a seizure, convulsive, or nonconvulsive, can harm vulnerable tissues, such as the ischemic penumbra [13]. Early recognition and treatment of seizures may prevent damage to the blood-brain barrier and improve recovery and overall outcome [9–13]. A challenge to this treatment strategy is identifying seizures in stroke patients without clinical signs. This furthers the importance of continuous electroencephalography (cEEG) to diagnose electrographic nonconvulsive seizures [14].

Predictors of seizure recurrence in ischemic stroke patients who have ASyS are not well characterized. We present a five-year retrospective study of ischemic stroke patients who had ASyS during hospitalization, identifying risk factors for the development of seizure recurrence.

## 2. Methods

### 2.1. Patients

A retrospective review of consecutive patients aged 18 years or older admitted for ischemic stroke over a 60 month period was conducted. Data were retrieved from the epilepsy database (Ebase) and the electronic medical record (EMR). All patients with cEEG seizures following either acute (≤14 days) or remote (>14 days) ischemic stroke (IS) were identified, and only patients who had follow-up encounters in the electronic medical record were included in this review. cEEG at our center is generally placed on patients either due to an alteration in mental status or due to clinical suspicion for seizures. Hospital policy does not require prior authorization to order cEEG and, therefore, sensitivity to ASyS in our patient population is quite high. Patients with history of epilepsy or brain pathology outside of ischemic stroke were also excluded from this analysis.

Clinical data collected included patient demographics, location of stroke, level of consciousness at time of cEEG placement, discharge status, antiepileptic drug (AED) management during hospitalization and at follow-up, and the time from onset of seizures to follow-up appointment. The level of consciousness was defined as being awake (i.e., spontaneously eye opening), lethargic (i.e., eyes opening to minor stimulation), stuporous (i.e., eyes opening to stimulation but quickly closes), or comatose (i.e., no eye opening to stimulation). The discharge status of patients was simply classified as home or rehabilitation. The institutional review board approved this study.

### 2.2. Data Acquisition

Inpatient cEEG and outpatient routine EEG (i.e., 20 minutes of recording) were recorded using 21 electrodes placed according to the International 10–20 System by certified EEG technologists and interpreted by board-certified electroencephalographers. Seizures on cEEG were defined as evolving rhythms in frequency, distribution, and/or morphology at >2 Hz and for ≥10-second duration. Nonconvulsive seizure semiology included subtle movements, such as eye deviation, or if there were cEEG seizures without video or clinical findings. Nonconvulsive status was diagnosed if there was a continuous ictal pattern lasting >30 minutes or ictal pattern present in >50% of 1 hour of cEEG. Discharges on the cEEG were characterized as generalized periodic discharges (GPDs) with or without triphasic morphology, lateralized periodic discharges (LPDs), continuous slowing/background slowing, burst suppression, seizure, and/or status epilepticus.

### 2.3. Data Analysis

Data were summarized with descriptive statistics including means and standard errors of the mean (continuous data) and frequencies (categorical data). Categorical data were analyzed using Fisher's exact test. Odds ratios (OR) and 95% confidence interval (CI) analyses were performed using the Baptista-Pike test. Continuous data were analyzed using *t*-test between the groups. A *p* value of ≤0.05 was considered significant. Data were analyzed using GraphPad Prism 7 (LaJolla, CA, USA).

### 2.4. Data Availability

Anonymized data relevant to this study will be shared by request with any qualified investigator pending appropriate IRB approval(s).

## 3. Results

During the study period, 109 patients were diagnosed with ischemic stroke. Sixty-five patients (35M, 30F) ultimately met the inclusion criteria for this study. The average age (±SEM) of the patients was 61.9 ± 2.4 years.

### 3.1. Inpatient Data/Early Onset Seizures

#### 3.1.1. Acute vs Remote Stroke

Among the 65 patients, 27 (41.5%) presented with seizures in the acute ischemic stroke time period. The remaining 38 (58.5%) patients had seizures in the remote ischemic stroke time period.

#### 3.1.2. Continuous EEG

**Figure 1:**
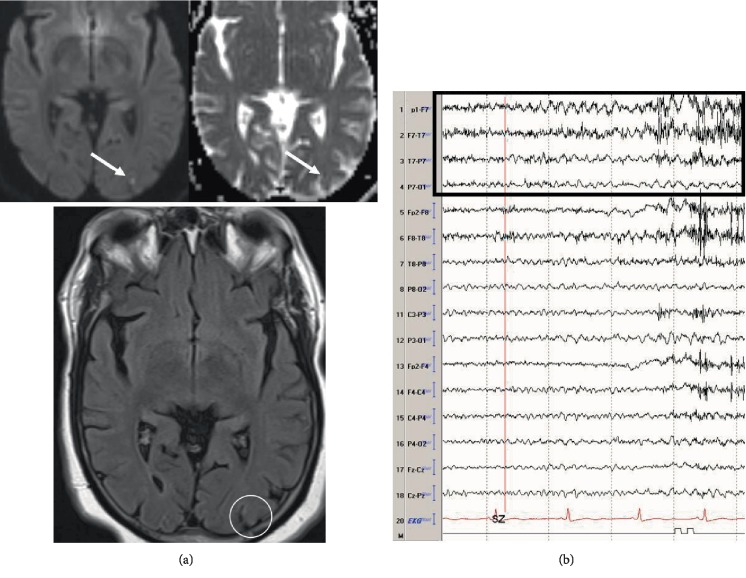
(a) MRI of the brain showing restricted diffusion on diffusion weighted imaging and corresponding decrease on apparent diffusion coefficient sequence (arrows, top images) and subtle hyperintensity on fluid attenuated inversion recovery sequence (circle, bottom image). (b) Bitemporal montage (5 second epoch, 50 *μ*v, sn 7 *μ*V/mm; LFF 1 Hz, HFF 70 Hz) showing a left temporal seizure (black box).

All of the patients identified with ASyS had nonconvulsive seizures on cEEG.  Table 1 summarizes the cEEG patterns. A majority (58/65, 89.2%) of the cEEG epileptogenic patterns seen during the initial hospitalization were epileptic seizures not meeting criteria for status epilepticus and closely followed by continuous slowing (54/65, 83.1%). Fourteen (14/65, 21.5%) patients showed LPDs on cEEG with 22.2% acute ischemic stroke and 21% remote ischemic stroke. An illustrative example of neuroimaging and EEG seizure in a patient is shown in Figure 1.

#### 3.1.3. Level of Consciousness

Twenty-nine patients were categorized as awake/lethargic, and the remaining thirty-six patients as comatose/stuporous.

#### 3.1.4. AEDs on Discharge

All the patients identified in this study were discharged on at least one AED (median of 2, range 1–4; Table 1). The most commonly prescribed AED was levetiracetam (80%) followed by phenytoin (41.5%). The majority (96.5%) of patients were discharged to a rehabilitation facility. Only one patient with an acute ischemic stroke and subsequent ASyS was discharged home.

### 3.2. Follow-Up/Late Onset Seizures

#### 3.2.1. Recurrence

After a mean (±SEM) follow-up of 358.2 ± 44.4 days, nineteen (19/65, 29.2%) of the patients in our study population were identified as suffering a clinical seizure recurrence. Sixteen (16/19, 84.2%) of the patients with seizure recurrence had a remote ischemic stroke at the time of their initial seizure, compared to three (3/19, 15.8%) who suffered acute ischemic stroke at the time of their first seizure (*p* = 0.0116, OR 0.17, 95% CI 0.049–0.65).

#### 3.2.2. EEG

A total of 31 patients from our study population had a follow-up routine EEG 384.4 ± 69 (mean ± SEM) days after their initial EEG. These EEGs were completed as part of follow-up visitation. A majority (11/31, 35.5%) of the EEG patterns seen at follow-up were classified as continuous slowing, closely followed by intermittent slowing (9/31, 29%; Table 2). 12.9% of the follow-up EEGs had sharp waves and/or spikes. The finding of sharp waves or spikes on routine EEG had a significant correlation with seizure recurrence (*p* = 0.006, OR 0, 95% CI 0–0.3926).

#### 3.2.3. Level of Consciousness and Seizure Recurrence

Twelve of the 36 patients (12/36, 33.3%) originally characterized as comatose/stuporous at the time of ASyS were diagnosed with seizure recurrence compared to seven of 29 (24.1%) who were classified as awake/lethargy while suffering from ASyS (*p* = 0.58).

#### 3.2.4. AED at Follow-Up

At follow-up, the majority of patients in our study group (57/65, 87.7%) remained on at least one AED (median 2, range 0–4). Only eight patients (8/65, 12.3%) were no longer on an AED. Two of these 8 patients suffered from recurrent seizures. Patients that were on ≥3 AEDs at follow-up, had a significant correlation with seizure recurrence (*p* = 0.0015, OR 0.05, 95% CI 0.0089–0.37; Table 1). No difference was found with level consciousness and AED polypharmacy between those without and with seizures at follow-up (*p* = 0.5).

## 4. Discussion

This retrospective study identifies predictive variables for recurrent seizures in ischemic stroke patients who had ASyS. We identified increased incidence of recurrent seizures in patients with remote ischemic stroke (i.e., >2 weeks after stroke onset), number of AEDs prescribed (≥3 AEDs), and sharp waves/spikes found on follow-up routine EEG. Additionally, since all patients had nonclinical seizures, we highlight the utility of using cEEG monitoring in the hospital setting to identify patients with seizures.

Berges et al. demonstrated in a study of 159 patients that poststroke seizures were frequent over a mean follow-up period of 47 weeks [15]. The Oxfordshire community stroke project followed up stroke patients with seizures for the recurrence of seizures [6]. They observed seizures in 2% of acute stroke patients (within 24 hours) [6]. Of these patients, 40% developed recurrent seizures. Likewise, in the Stockholm Incidence Registry of Epilepsy, the odds ratio for development of epilepsy after an ischemic stroke was 9.4 (95% CI 6.7–13.1) [16]. The identification of nonclinical seizures post ischemic stroke is becoming more recognized. In the study by Belcastro et al., they found that 3.6% of all stroke patients had nonclinical seizures [17]. The risk of nonclinical seizures is great in the first 7 days following an ischemic stroke [18]. During this time, ~20% of seizures are nonclinical [18]. Our data are consistent with these studies showing seizure recurrence after stroke to occur in 29.2% of our cohort. The majority of seizures are nonconvulsive. Without a robust electrophysiological monitoring program, many of these seizures in our study would have gone unnoticed.

Defining the time period following ischemic stroke as acute or remote has been a matter of discrepancy in the literature. A review of 714 stroke patients (both hemorrhagic and ischemic) found that seizures after acute ischemic strokes occurred in 4.2% of patients within 14 days or less [19]. Likewise, we used the time point of 14  days or less as the time-point cutoff for acute ischemic stroke. Our study demonstrated that patients with ASyS following remote ischemic strokes (>2 weeks) tended to have a higher risk of developing seizure recurrence over a mean follow-up period of approximately 51 weeks. Additionally, the finding of sharp waves on follow-up routine EEG further identified those at risk of seizure recurrence. CEEG is becoming increasingly common for monitoring neurologically injured patients [20]. With longer monitoring, subclinical seizures are also increasingly recognized. Additionally, it appears clinically necessary to provide prolonged monitoring for patients that are comatose or stuporous [20].

There are a few population-based studies that attempted to determine the predictors of seizure based on localization and type of ischemic stroke. The Greater Cincinnati population based study found that patients with cardio-embolic ischemic strokes had a significantly higher incidence of seizures compared to the small or large vessel ischemic strokes [21]. The Oxfordshire community project found that large anterior circulation infarcts had a markedly increased risk of recurrent seizures compared to ischemic strokes affecting other vascular territories [6]. Graham et al. showed that the 10-year estimate of poststroke seizure recurrence from either total anterior circulation infarct, partial anterior circulation infarct, or posterior circulation infarct was 28.7%, 13.4%, and 4.8%, respectively [22]. Studies have found that ischemic strokes involving the cortex, especially those with hemorrhagic transformation, were at a higher risk of seizures [19, 23–25]. Others have looked at such as NIH stroke scale (NIHSS), infarct size, and etiology of the ischemic stroke [17]. However, NIHSS has mixed results. A previous study found it to not be an independent predictor of early seizure after stroke [23]. A more recent paper suggests that an NIHSS of >4 along with prolonged seizures (i.e., status epilepticus) may increase risk of post stroke epilepsy [26]. Recently a group has created the SeLECT score based on severity of stroke, large-artery atherosclerosis, early seizure (<7 days), cortical involvement, and territory of MCA [27]. Being able to risk stratify patients is becoming recognized as necessary. Unfortunately, the data used in these algorithms can be conflicting with current literature.

When patients have poststroke seizures, the choice and number of AEDs is variable. Belcastro et al. demonstrated a possible neuroprotective role of levetiracetam in brain ischemia, and suggested that it is safe and effective in poststroke seizures [28]. A large nationwide, population-based study in Taiwan illustrated that using valproate (VPA) and newer AEDs had better seizure control than phenytoin (PHT) in patients with late onset seizure recurrence [29]. The endpoints of this study were lower number of emergency department visits and hospitalization [29]. Studies have shown that long-term exposure to older agents, such as PHT, can lead to poorer neuropsychological outcomes [30, 31]. On the other hand, PHT usage in epilepsy has been shown to attenuate the stroke risk [32]. Moreover, the choice of AED in the elderly is complicated by the side-effect profile of AEDs and altered metabolism and clearance in the elderly [7]. Overall, there is a lack of evidence to support any one particular AED for prevention of seizure recurrence [12]. The choice of AED should be individualized to the patient. In our study, patients who required a greater overall number of AEDs to control their seizures in the hospital were at the highest risk of having recurrent seizures. This highlights the severity of the initial acute symptomatic seizures and need for a closer follow-up post hospital discharge in patients on multiple AEDs.

This study by nature of being a retrospective chart review has limitations inherent in data collection. Additionally, patients included in this study had a 60.5% follow-up rate for EEG. Our sample size is somewhat small in each EEG pattern group which may be the reason for the negative findings in the predictive value of cEEG results for recurrent seizures. Future studies need to evaluate several important questions in the treatment of seizure after ischemic strokes, including whether or not ASyS, particularly nonconvulsive seizures, worsen ischemic stroke; if aggressive treatment of nonconvulsive seizures improves or changes outcome; which AED portends the best long-term functional outcome if seizures are treated; and whether or not initial patient severity of illness influences seizure burden.

## 5. Conclusion

Patients with remote ischemic strokes (>2 weeks), on multiple AEDs (≥3 AEDs) at follow-up, and those with sharp waves/spikes on follow-up EEG have an increased seizure recurrence potential if they suffered from ASyS at initial presentation.

## Figures and Tables

**Table 1:** Patient demographics at follow-up.

	Recurrent seizures		No recurrent seizures		*p* value
*Demographics*					
Total	19		46		NS
Age (yrs, avg, SEM)	56.9	18.2	64	21.9	NS
Female	12	63.2	18	39.1	NS
Coma/stupor	12	63.2	24	52.2	NS

*Ischemic stroke*					
Acute	3	15.8	24	52.2	0.01
Remote	16	84.2	22	47.8	

*Outcome*					
Discharged home	5	26.3	6	13.0	NS
Discharged rehab	14	73.7	40	87.0	NS

*Number of AEDs on initial hospital discharge*					
0	0	0	0	0	NS
1	5	26.3	19	41.3	NS
2	6	31.6	22	47.8	NS
>3	8	42.1	5	10.9	0.01

*Number of AEDs at F/U appointment*					
0	2	10.5	6	13.0	NS
1	6	31.6	18	39.1	NS
2	6	31.6	17	37.0	NS
>3	5	26.3	5	10.9	0.002
Clinic follow-up (days, avg, SEM)	580.1	266.6	266.6	227.0	0.002

Abbreviations: *n*: number, yrs: years, AEDs: antiepileptics, F/U: follow-up, NS: nonsignificant, avg: average, SEM: standard error of the mean. Data expressed as *n*, % unless otherwise specified.

**Table 2:** CEEG findings on CEEG in hospital and on routine EEG at outpatient follow-up.

	Recurrent seizures		No recurrent seizures		*p* value
*Initial EEG*					
Total	19		46		
Status epilepticus	6	31.6	11	23.9	NS
Epileptic seizures	17	89.5	41	89.1	NS
GPDs with triphasic waves	0	0	0	0	NS
LPDs	3	15.8	11	23.9	NS
GPDs	0	0	7	15.2	NS
Continuous slow	15	78.9	39	84.8	NS
Burst suppression	0	0.0	2	4.3	NS

*Follow-up EEG*					
Total	10		21		
Normal	0	0	4	19.0	NS
Epileptic seizures	3	30.0	0	0	0.01
Sharp wave/spike	4	40.0	0	0	0.006
GPDs with triphasic waves	0	0.0	0	0	NS
LPDs	1	10.0	0	0	NS
GPDs	0	0	0	0	NS
Continuous slow	2	20.0	9	42.3	NS
Background slow	1	10.0	0	0	NS
Intermittent slow	2	20.0	7	35.3	NS
Time to follow-up (days, avg, SEM)	506.8	397.7	317.1	586.5	NS

Abbreviations: CEEG: continuous electroencephalogram, LPDs: lateralized periodic discharge, GPDs: generalized periodic discharges, EEG: electroencephalogram, *n*: number, SEM: standard error of the mean. Data expressed as *n*, % unless otherwise specified.

## Data Availability

Anonymized data relevant to this study will be shared by request with any qualified investigator pending appropriate IRB approval(s).
